# Reduced red and processed meat consumption is associated with lower diet costs in US households: a national analysis of protein substitutions

**DOI:** 10.1017/S1368980024001897

**Published:** 2024-10-10

**Authors:** Dania Orta-Aleman, Andrew L Thorne-Lyman, Roni Neff, Julia Wolfson, Laura E Caulfield

**Affiliations:** 1 Nutrition Policy Institute, University of California Agriculture and Natural Resources, 1111 Franklin Street, 11th Floor, Oakland, CA 94607, USA; 2 Center for Human Nutrition, Johns Hopkins Bloomberg School of Public Health, 615 N. Wolfe Street, Baltimore, MD 21205, USA; 3 Center for a Livable Future, Department of Environmental Health & Engineering, Johns Hopkins Bloomberg School of Public Health, 615 N. Wolfe Street, W7009, Baltimore, MD 21205, USA; 4 Department of Environmental Health & Engineering, Johns Hopkins Bloomberg School of Public Health, 615 N. Wolfe Street, W7009, Baltimore, MD 21205, USA; 5 Department of International Health, Johns Hopkins Bloomberg School of Public Health, 615 N Wolfe Street, Baltimore, MD 21205, USA; 6 Department of Health Policy and Management, Johns Hopkins Bloomberg School of Public Health, Baltimore, MD 21205, USA

**Keywords:** Red meat, Processed meat, Protein, Dietary cost, Food expenditures

## Abstract

**Objective:**

To examine the association between red and processed meat consumption and total food expenditures in US households and explore whether households could reduce food costs by substituting these meats with other protein sources such as poultry, seafood, eggs and plant proteins.

**Design:**

Cross-sectional study using data from the National Household Food Acquisition and Purchase Survey (FoodAPS). Using adult male equivalents (AME) for standardisation, we categorised red and processed meat purchases into quintiles. We used generalised linear models to explore the association between red or processed meat consumption and food expenditures and the cost effect of substituting meat with other proteins.

**Setting:**

United States.

**Participants:**

Data from 4739 households with valid acquisition information from FoodAPS, a stratified multistage probability sample of US households.

**Results:**

Higher red and processed meat consumption were both significantly associated with higher total weekly food expenditures, particularly among households with low income. Substituting red or processed meat with poultry, eggs or plant proteins did not significantly affect overall food expenditures, whereas replacing meat with seafood, especially varieties high in *n*-3 fatty acids, led to increased costs.

**Conclusions:**

Reducing red and processed meat consumption could offer savings for households, particularly those with low income. Although substitutions with seafood high in *n*-3 could increase expenses, alternative protein sources like poultry and plant proteins may serve as cost-neutral replacements. Public health strategies should emphasise dietary shifts’ economic, health and environmental benefits and aim to make nutritious yet affordable protein sources more accessible.

High red and processed meat consumption has been associated with a higher risk of all-cause mortality^(1)^ and chronic diseases, including CVD^(1–4)^ and colorectal, stomach and pancreatic cancer^(5,6)^. Conversely, consumption of poultry, fish, nuts and plant proteins has been associated with lower mortality and chronic-disease risk, particularly when they replace red and processed meats^(7,8)^. Reducing meat consumption may also benefit the environment by reducing greenhouse gas emissions, deforestation, water use and biodiversity loss^(9–11)^. Accordingly, the Intergovernmental Panel on Climate Change has recommended reducing meat consumption for health and environmental reasons^(12)^. Despite these recommendations, the economic implications of reducing or substituting red and processed meat consumption have not been widely studied.

In the USA, one of the highest per capita consumers of meat globally, meat significantly contributes to the average diet, accounting for 15 % of daily energy intake, 40 % of daily protein intake and 20 % of daily fat intake^(13–15)^. The impact of such high meat consumption on household food budgets is important to understand, as affordability influences food choices, especially among households with low income who devote a larger share of their income to food^(16,17)^. Although healthier foods and diets are typically more expensive^(18–22)^—potentially exacerbating health disparities—meat is a costly protein source, suggesting that reducing its consumption could lower overall diet costs^(23,24)^. However, detailed studies on the specific economic implications of varying levels of meat consumption are scarce.

Moreover, while the overall protein intake in the US generally meets minimum requirements, there are significant gaps in the consumption of specific recommended proteins^(25)^. Nearly 90 % of the US population does not meet seafood intake recommendations, about 80 % fall short of legume intake and over half do not meet the recommended intake of nuts, seeds and soy products^(25)^. Despite frequent recommendations to substitute red and processed meats with these healthier alternatives to improve diet quality^(26)^, the potential costs associated with such dietary changes are often overlooked in the literature. This study aims to fill this gap by evaluating the cost implications of adopting some of these recommended dietary changes, providing insights into the economic feasibility of shifting towards healthier, recommended protein sources.

Previous studies have often relied on imputation methods to estimate the costs of meat items based on national food prices, which may not accurately reflect the prices consumers pay or account for regional variations^(27–30)^. Moreover, these studies have not differentiated between types of meat (e.g. processed meat *v*. unprocessed meat; poultry *v*. red meat) or examined the cost implications of substituting meat with other protein sources^(27–30)^.

The objectives of our study are twofold: (i) to examine the association between red and processed meat consumption and total food expenditures in a nationally representative sample of US household purchases and (ii) to explore the effect of substituting these meats with equivalent amounts of other protein sources such as poultry, seafood, eggs and plant proteins on food expenditures. Utilising data from the National Household Food Acquisition and Purchase Survey (FoodAPS), we hypothesised that higher red and processed meat consumption is associated with increased food expenditures and that substituting these meats meat with poultry, eggs or plant proteins will lower overall food expenditures while substituting with seafood will increase them.

## Methods

### Study sample

We analysed FoodAPS data, a nationally representative cross-sectional survey of US households’ food purchases and acquisitions conducted by the US Department of Agriculture’s Economic Research Service (USDA-ERS) between April 2012 and January 2013^(31)^. FoodAPS used a stratified multistage probability sample design to collect detailed food purchases and acquisition information for both at-home and away-from-home consumption across 4826 households.

The data included barcode scans and receipts for packaged foods, identification of food-at-home and foods-away-from-home purchases, item quantities and sizes, prices, expenditures, place of acquisition and payment type, as well as household and individual socio-demographic characteristics. All household members aged 11 years or older were asked to record their food purchases and acquisitions in designated food booklets for seven consecutive days. We included 4739 households with valid acquisition data in our analysis, excluding 87 households that did not record any purchases or acquisitions.

### Measures

#### Outcome and exposure variables

The outcome variable was a continuous measure representing weekly household food spending in US dollars, calculated by summing all reported food spending for at-home and away-from-home consumption.

The main exposure variables were weekly red meat purchases in ounce equivalents (oz. eq.) per adult male equivalent (AME) and weekly processed meat purchases (oz. eq.) per AME. Purchased food was categorised by the USDA-ERS into broad food categories based on their ingredients, preparation methods and nutritional profiles^(32)^. Nutrition information and food pattern equivalents were matched to each item using the USDA National Nutrient Database for Standard Reference (SR28) and the USDA Food Patterns Equivalents Database (FPED) 2011–2012. We added all foods in the red meat or processed meat categories according to the USDA FPED^(32)^. We multiplied the energy and food pattern ounce equivalents by the edible gram weight of each food using a previously described methodology and the imputed missing quantities provided by the ERS^(32)^. This method accounts for both single-ingredient items (e.g. raw chicken breast) and mixed dishes (e.g. chicken pot pie) by disaggregating them into their component ingredients and applying appropriate yield factors and cooking losses.

Red meat included beef, veal, pork, lamb, organ meats and cured red meat. Processed meat included any meat preserved by smoking, curing, salting or adding chemicals, including processed red meat (e.g. ham, bacon) and processed poultry meat (e.g. turkey deli meat). We categorised both exposure variables into quintiles, adjusting for FoodAPS survey weights. Similarly, for the substitution analysis, we identified and categorised other high protein foods such as poultry, fish and seafood high in *n*-3 fatty acids, fish and seafood low in *n*-3 fatty acids, plant proteins (including nuts, peanuts, seeds, legumes and soy products), and eggs. We report all meat purchases as grams and as ounce equivalents (oz. eq.). Ounce equivalents (oz. eq.) is a standard unit of measurement that allows for standardised comparison across different protein sources^(33)^. One ounce equivalent represents 28 grams of meat, poultry or fish; 1/4 cup of cooked beans, peas or lentils; 1 egg; 1 tablespoon of peanut butter or 14 grams of nuts or seeds. See details about meat and other protein food classifications in online Supplementary Table S1.

To account for household size and composition differences, we calculated AME, considering the estimated energy requirements (EER) by age and sex and considering a sedentary physical activity level for each household member, as found in the Dietary Guidelines for Americans^(34)^. This method adjusts consumption relative to nutritional needs and avoids the problem of per capita purchases, where a child counts the same as an adult.

We included several covariates in our analysis that could confound the association between red or processed meat purchases and total food expenditures. We selected these covariates based on previous literature showing their association with meat consumption and food expenditures^(35,36)^. We calculated total purchased energy per AME (kilojoules/AME) by aggregating the caloric value of all food acquisitions in the household and dividing by AME. Other covariates included household size, rurality, household poverty-income ratio (PIR), geographical area (Northeast, Midwest, South, West), number of children in the household, number of adults over 60, participation in the Supplemental Nutrition Assistance Program (SNAP), the proportion spent on food-away-from-home. Primary respondent characteristics included sex, age (available as categories for confidentiality: 18–35 years, 36–59 years, more than 60 years), race and ethnicity (Non-Hispanic White, Non-Hispanic Black, Non-Hispanic other race, Hispanic), education (high school, some college, bachelor’s degree or more) and being married or living with a partner. A household with low income was defined as having a PIR below 130 % of the federal poverty line.

### Statistical analysis

We used FoodAPS sampling weights to account for the survey’s complex sampling design. First, we examined the socio-demographic characteristics across weighted quintiles of weekly red meat and processed meat purchases. For the first part of our analysis, to examine the association between red and processed meat consumption and total food expenditures, we used a survey-adjusted generalised linear model (GLM), using a gamma distribution for weekly food spending and a log link to covariates due to the skewed distribution of weekly spending and the strictly positive continuous values of the outcome variable^(37,38)^. We tested the distribution of weekly food spending and confirmed that the gamma distribution was appropriate for our data.

The main exposure variables in all models were categorical variables indicating the weighted quintiles of weekly red meat purchases per AME and weekly processed meat purchases per AME to which a household was assigned. The third quintile was set as the reference. We adjusted for total purchased energy per AME to analyse the relative effect of red or processed meat purchases on total food expenditures for the same total energy level^(39)^.

We built five sequential models to understand how different categories of factors may confound the association between red or processed meat purchasing and total household food expenditures. Model 1 showed the crude association. Model 2 adjusted for household size and the number of children. Model 3 adjusts for model 2 plus PIR and SNAP participation. Model 4 adjusts for all previous variables plus geographical area, rurality, characteristics of the primary respondent (sex, age, education, race and ethnicity, marital status) and the proportion of foods-away-from-home. Model 5 included all previous variables plus the total purchased energy per AME.

We reported model results as exponentiated beta values, interpreted as multiplicative effects on total weekly food spending. We also reported differences in US dollars spent on food per week to facilitate interpretation using Stata’s post-estimation predictive margins.

To test whether the association between purchases of red and processed meat and total expenditures vary by income level, we tested for effect-measure modification between the highest quintile of meat purchasing and a household with low income (PIR below 130 % federal poverty line) by including a cross-product term in Model 5. This interaction term captures the joint effect of being in the highest quintile of meat purchasing and having a low income on total food expenditures. We chose this interaction term because we hypothesised that low-income households may be more sensitive to the cost of meat products.

Finally, for the second part of our analysis, we investigated the association of substituting 28·3 g (1 oz. eq.) of red or processed meat with an equivalent amount of other protein foods, including poultry, seafood, plant proteins and eggs, on total food expenditures. To create the substitution model, we simultaneously included both protein items as continuous variables in the multivariable model described above (Model 5). We included total energy per AME to assess the association between substitutions independent of total energy purchases. Following nutritional epidemiology substitution modelling recommendations, we calculated the substitution effect by deriving the difference between their respective coefficients, reflecting the marginal effects of increasing or decreasing one protein item while holding all other variables constant^(40)^.This approach allowed us to quantify the association between increased consumption of specific alternative protein foods and a corresponding reduction in red or processed meat intake while maintaining the same overall energy intake level. The methodology aligns with substitution modelling techniques described in more detail elsewhere^(40)^.

We conducted the analyses using Stata se, Version 15.

## Results

Table 1 shows socio-demographic characteristics by weighted quintile of weekly red meat purchases. Households in the highest quintile (Q5) of red meat purchases acquired a weekly average of 1744·7 g per AME (61·5 oz. eq. per AME), compared to 25·9 g per AME (0·9 oz. eq. per AME) in the lowest quintile (Q1). Households purchasing the highest amount of red meat (Q5) had a smaller size, lower income and PIR, fewer children, more adults over 60, were more likely to reside in the South and rural areas and had higher SNAP participation. Respondents in Q5 were likelier to be female, older, non-Hispanic White and have lower educational attainment than respondents in Q1–4.

**Table 1:** Socio-demographic characteristics of FoodAPS US households by quintile of weekly red meat purchases

Weighted quintile of weekly red meat purchases per AME															
	Q1			Q2			Q3			Q4			Q5		
	%	Mean	sd	%	Mean	sd	%	Mean	sd	%	Mean	sd	%	Mean	sd
Red meat (g) per AME		25·9	1·7		155·4	1·8		333·9	3·2		613·9	5·7		1744·7	78·1
Household size		2·2	0·07		2·6	0·05		2·6	0·07		2·6	0·07		2·1	0·05
Household income		4621	335		5040·7	202		5458	258		5113	249		4169·2	183
Mean PIR		376·5	24		369·4	12·7		411·3	25·1		375·5	15·2		338·3	13·9
Income, % of FPL															
< 130 %	20·6			17·0			13·9			15·6			21·8		
130–349 %	37·5			42·1			41·7			41·3			41·4		
≥ 350 %	42·0			40·9			44·4			43·1			36·8		
# children < 18 years		0·5	0·04		0·74	0·03		0·69	0·04		0·66	0·03		0·43	0·03
# of adults > 60		0·5	0·03		0·4	0·03		0·4	0·03		0·5	0·02		0·6	0·04
SNAP participation	13·7			13·7			11·9			10·1			17·0		
Geographical area															
Northeast	18·2			16·3			15·7			14·5			12·5		
Midwest	28·8			28·9			33·2			31·4			34·8		
South	34·6			34·0			31·9			36·2			40·3		
West	18·4			20·9			19·2			17·9			12·4		
Rurality	30·2			29·0			31·5			35·8			43·6		
Primary respondent															
Female (%)	65·1			67·5			68·7			67·2			70·1		
Married (%)	34·8			45·0			48·3			50·5			44·7		
Age (%)															
18–35 years	27·3			34·4			22·6			21·3			14·0		
35–59 years	42·0			40·4			50·8			50·1			46·0		
60 years and over	30·7			25·2			26·6			28·6			40·1		
Education level (%)															
High school or less	36·4			27·2			29·7			34·0			42·6		
Some college	29·9			36·1			36·9			31·9			32·6		
Bachelor’s degree+	33·7			36·7			33·4			34·1			24·8		
Race/ethnicity (%)															
NH-White	63·5			64·4			70·1			72·2			72·9		
NH-Black	16·2			12·8			9·4			10·9			10·6		
NH-Other	8·3			6·5			8·0			5·9			4·1		
Hispanic	12·0			16·3			12·5			11·0			12·5		
Healthy food costs too much	33·0			34·2			35·6			31·3			30·3		

AME, adult male equivalents; PIR, poverty-income ratio; FPL, federal poverty line; SNAP, Supplemental Nutrition Assistance Program; HH: household; NH-White: non-Hispanic White; Q: quintile..

Table 2 shows that households in the highest quintile of weekly processed meat purchases (Q5) acquired an average of 1314·7 g (46·4 oz. eq.) of processed meat per AME, whereas households in the lowest quintile acquired 207·2 g (7·3 oz. eq.). Compared to those in Q1–4, households in Q5 of processed meat had lower incomes, were older, more educated, predominantly female and non-Hispanic White and were more likely enrolled in SNAP compared with those in Q1–4. In contrast, households in the lowest quintile of processed meat were smaller, more likely to have an income below 130 % of the federal poverty line and be non-Hispanic Black and less likely to be married and non-Hispanic White compared to households in Q2–5.

**Table 2:** Socio-demographic characteristics of FoodAPS US households by quintile of weekly processed meat purchases

Weighted quintile of weekly processed meat acquisitions per adult male equivalent (AME)															
	Q1			Q2			Q3			Q4			Q5		
	%	Mean	sd	%	Mean	sd	%	Mean	sd	%	Mean	sd	%	Mean	sd
Processed meat(g) per AME		207·2	27·9		314·4	30·0		421·0	23·0		615·8	28·7		1314·7	81·3
Household size		2·1	0·04		2·6	0·05		2·7	0·06		2·6	0·05		2·2	0·05
Household income		4430·4	249·1		5083·2	301·2		5381·9	216·5		5320·7	213·5		4188·7	194·6
PIR		371·1	17·7		378·4	23		397·4	14·3		400·1	16·4		324·1	11·8
Household income as % FPL															
< 130 %	22·5			16·0			14·6			14·9			20·8		
130–349 %	35·5			43·5			39·9			41·1			44·2		
≥ 350 %	42·0			40·6			45·5			44·0			35·0		
# of children < 18		0·4	0·03		0·7	0·04		0·7	0·04		0·7	0·04		0·5	0·04
# of adults > 60		0·5	0·03		0·4	0·04		0·4	0·04		0·5	0·03		0·6	0·03
SNAP participation	13·0			13·2			12·5			12·7			15·0		
Geographical area															
Northeast	16·3			16·9			15·8			14·8			13·6		
Midwest	26·4			28·6			33·0			34·6			34·3		
South	37·1			34·8			30·8			33·9			40·3		
West	20·2			19·7			20·4			16·7			11·8		
Rurality	31·1			31·7			32·6			30·9			43·8		
Primary respondent															
Female (%)	61·9			62·8			68·8			69·4			75·8		
Married (%)	35·2			44·1			47·2			53·2			43·6		
Age (%)															
18–35 years	23·3			31·9			25·3			22·5			16·6		
35–59 years	38·3			44·4			51·9			49·0			45·7		
60 years and over	38·4			23·7			22·8			28·5			37·7		
Education level (%)															
HS or less	36·8			30·6			32·1			30·9			39·4		
Some college	27·4			37·3			32·1			36·9			34·0		
Bachelor’s or +	35·9			32·1			35·8			32·3			26·7		
Race/ethnicity (%)															
NH-White	61·4			68·0			65·0			70·2			78·5		
NH-Black	13·9			14·7			9·7			11·9			9·8		
NH-Other	9·7			7·2			7·7			4·7			3·2		
Hispanic	14·9			10·1			17·6			13·1			8·6		

AME, adult male equivalents; PIR: poverty–income ratio; FPL, federal poverty line; SNAP, Supplemental Nutrition Assistance Program; HS: high school; HH: household; NH-White: Non-Hispanic White.

Table 3 presents our GLM models for the association between the weighted quintile of red and processed meat purchases and weekly household food spending. The unadjusted model (Model 1) shows a dose–response association: compared to Q3, the two highest quintiles of red meat purchases were associated with higher food expenditures and lower quintiles with lower food expenditures. All associations remained significant after adjusting for household size and composition (Model 2), income variables (Model 3) and geography, rurality and socio-demographic characteristics of the primary respondent (Model 4). After adjustment for energy per AME, the associations attenuated but remained significant (Model 5).

**Table 3:** Exponentiated coefficients of weekly FoodAPS US household food expenditures by quintile of red and processed meat purchases

	Model 1		Model 2		Model 3		Model 4		Model 5	
	Unadjusted model		Adjusted by HH size and # of children and adults > 60 in the HH		Model 2 + poverty ratio and SNAP participation		Model 3 + geography, rurality, FAFH and character of PR		Model 4 + weekly energy per AME ^a^	
	e^*β*^	95 % CI	e^*β*^	95 % CI	e^*β*^	95 % CI	e^*β*^	95 % CI	e^*β*^	95 % CI
Weighted quintile of weekly red meat purchases per AME										
Q1	0·54	0·49, 0·6	0·63	0·56, 0·72	0·65	0·58, 0·73	0·66	0·6, 0·74	0·77	0·69, 0·87
Q2	0·80	0·7, 0·91	0·82	0·74, 0·92	0·83	0·75, 0·92	0·83	0·75, 0·91	0·90	0·81, 0·99
Q3	1	(Ref)	1	(Ref)	1	(Ref)	1	(Ref)	1	(Ref)
Q4	1·19	1·1, 1·29	1·20	1·1, 1·31	1·20	1·12, 1·3	1·21	1·12, 1·3	1·15	1·08, 1·22
Q5	1·16	1·06, 1·26	1·32	1·21, 1·45	1·40	1·29, 1·52	1·47	1·35, 1·6	1·16	1·05, 1·29
Weighted quintile of weekly processed meat purchases per AME										
Q1	0·60	0·51, 0·71	0·69	0·59, 0·81	0·72	0·63, 0·82	0·71	0·63, 0·81	0·75	0·65, 0·86
Q2	0·79	0·7, 0·9	0·79	0·72, 0·88	0·83	0·75, 0·92	0·82	0·74, 0·89	0·81	0·74, 0·89
Q3	1	(Ref)	1	(Ref)	1	(Ref)	1	(Ref)	1	(Ref)
Q4	1·08	0·99, 1·18	1·08	1, 1·16	1·11	1·04, 1·18	1·09	1·01, 1·18	1·08	1·01, 1·17
Q5	1·20	1·04, 1·38	1·26	1·12, 1·41	1·34	1·22, 1·47	1·37	1·26, 1·48	1·42	1·31, 1·54

HH: household; ref: reference; SNAP: Supplemental Nutrition Assistance Program; FAFH, foods-away-from-home; PR: primary respondent; AME, adult male equivalents.

Model 5: adjusted regression estimates from generalised linear model with gamma distribution and log link, incorporating FoodAPS strata and sampling weights, adjusted for household size, number of children in the household, number of adult members over 60 in the household, household income to poverty ratio, SNAP participation, geographical area, rurality, proportion of food-away-from-home *v*. food-at-home, primary respondent demographic characteristics (sex, age, race/ethnicity, education and being married) and weekly energy per AME.

The fully adjusted model (Model 5) showed a positive relationship between the weighted quintile of weekly red meat purchases and household food expenditures. After adjustment for energy and other covariates, households on the highest quintile of red meat purchasing spent 16 % (e^*β*^ = 1·16; 95 % CI: 1·05, 1·29) more on food per week than households in the middle quintile of red meat consumption and 51 % more than households in the lowest quintile (See online Supplementary Table S2). In terms of dollars, on average, a household in Q5 of red meat consumption spent $43 more than a household in the middle quintile after adjusting for covariates (See Table 4).

**Table 4:** Estimated US dollar differences in weekly household food expenditures compared with the lowest level of meat consumption in oz. eq

Weighted quintile of weekly red meat purchases (95 % CI)^a^		
Q1	−24·2	−31·7, –16·6
Q2	−12·6	−19·6, –5·7
Q3	Ref	
Q4	20·5	13·4, 27·7
Q5	43·5	33·4, 53·5
Weighted quintile of weekly processed meat purchases (95 % CI)		
Q1	−21·9	−31·2, –12·5
Q2	−16·4	−23·5, –9·4
Q3	Ref	
Q4	7·0	0·1, 14·0
Q5	36·5	27·3, 45·8

oz. eq.: ounce equivalents; SNAP: Supplemental Nutrition Assistance Program.

a Estimated from fully adjusted regression estimates from generalised linear model with gamma distribution and log link, incorporating FoodAPS strata and sampling weights and adjusted for household size, rurality, household income to poverty ratio, geographical area, number of children in the household, number of adult members over 60 in the household, SNAP participation, proportion of food-away-from-home *v*. food-at-home, weekly energy per AME and primary respondent demographic characteristics including sex, age, race/ethnicity, education and being married.

Processed meat findings mirrored red meat, with the highest quintiles being associated with higher total food expenditures and the lowest quintiles being associated with lower food expenditures in Models 1–4. After adjustment for weekly energy per AME (Model 5), the negative association between the fourth quintile attenuated but remained significant. Households on the highest quintile of processed meat purchasing spent 42 % (e^*β*^ = 1·42; 95 % CI: 1·31, 1·54) more on food per week than households in the middle quintile of processed meat consumption, and 33 % more than households in the lowest quintile of processed meat consumption, corresponding to an additional $36 per week.

We tested for effect modification by income level and found a significant interaction term (*P* = 0·003), indicating that living in a low-income household was associated with a stronger positive association between high red meat purchases and weekly food expenditures.

**Fig. 1:**
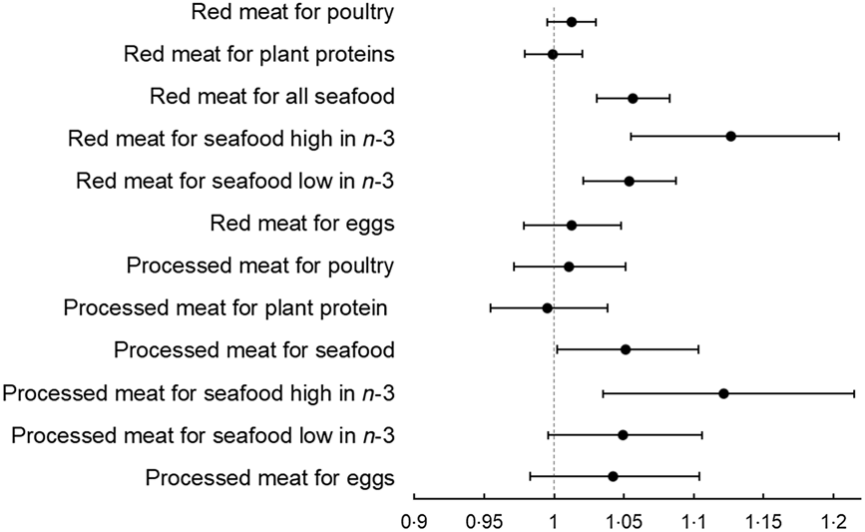
Adjusted exponentiated coefficients and 95 % CI for total diet cost associated with replacing 28·3 g (1 oz. eq.) per day of red or processed meat with an equivalent amount per day of another protein source. Generalised linear models with gamma distribution and log link adjusted for the following covariates household size, rurality, household income to poverty ratio, geographical area, number of children in the household, number of adult members over 60 in the household, SNAP participation, proportion of food-away-from-home and food-at-home, weekly energy per AME; and primary respondent demographic characteristics including sex, age, race/ethnicity, education and being married

Finally, for the second part of our analysis, we investigated the effect of substituting red or processed meat with other protein sources on total food expenditures. Substituting 28·3 g/d (1 oz. eq./d) of red meat with an equivalent amount of seafood was associated with a 6 % increase in total weekly food expenditures after multivariable adjustment (See Fig. 1). In particular, replacing 28·3 g/d (1 oz. eq./d) of red meat with an equivalent amount of seafood high in *n*-3 was associated with a 13 % increase in total weekly food expenditures. In contrast, replacing 28·3 g/d (1 oz. eq./d) of red meat with an equivalent amount of poultry, plant proteins or eggs was not significantly associated with increased total food expenditures.

For processed meat, replacing 28·3 g/d (1 oz. eq./d) with an equivalent amount of seafood high in *n*-3 fatty acids was associated with a 12 % increase in total weekly food expenditures. Substituting 28·3 g/d (1 oz. eq./d) of processed meat with an equivalent amount of poultry, plant protein, eggs or seafood low in *n*-3 fatty acids was not significantly associated with increased food expenditures.

## Discussion

This study examined the association between red meat and processed meat consumption and total weekly food expenditures in US households, and the effects of substituting these meats with alternative protein sources on food expenditures. Key findings include: (1) higher red and processed meat consumption were both significantly associated with higher food expenditures, with a dose-response relationship; (2) this association is more pronounced in households with low income and (3) substituting red or processed meat with seafood high in *n*-3 fatty acids was associated with increased food expenditures, whereas substitutions with poultry, plant proteins or eggs are cost-neutral.

Our findings align with prior studies showing a positive association between animal protein consumption and diet costs^(28,41)^. However, our results differ from a recent study suggesting that households purchasing moderate levels of red meat as a share of their total food budget spend the most on food per week compared with those in higher or lower quintiles^(42)^. This discrepancy may stem from issues derived from using ratio variables^(43)^, where the outcome (total weekly food spending) was also included as part of the exposure (spending in red meat/total weekly food spending). Our study avoids these methodological issues by using absolute quantities of red and processed meat per AME as the exposure variable.

The first part of our analysis reveals that households in the highest quintiles of red and processed meat consumption spend significantly more on food weekly compared with those in lower quintiles, even after adjusting for other covariates. This finding suggests that reducing consumption of these meats, rather than substituting them with other proteins, could result in cost savings. For instance, households in the highest quintiles of red and processed meat consumption could save up to $2236 and $1872 annually, respectively—a considerable portion of the average annual food expenditure of $6602^(44)^. However, cost may not be the primary driving factor behind the high consumption of these meats in the USA, as other factors such as preferences, habits, time and social influences may also play a role^(45,46)^.

Furthermore, our results highlight that households with low income may face greater financial burdens from consuming high amounts of red or processed meat than higher-income households with similar consumption levels. There could be multiple explanations for this finding. Households with low income may face higher prices due to regional food price variation or the retail place where they can purchase food^(47–51)^. Moreover, low-income households face barriers to buying bulk package sizes that can be more affordable^(51)^.

Despite the common perception that healthier foods are more expensive, the second part of the analysis, the substitution analysis, reveals that replacing red and processed meats with poultry, plant proteins or eggs can be cost-neutral. This finding challenges the notion that adhering to certain dietary guidelines necessitates higher expenditures. However, substituting these meats with seafood, particularly varieties rich in *n*-3 fatty acids, which offer significant health benefits, leads to higher costs. This could partly explain the low fish and seafood intake observed in the USA, especially among low-income populations^(52,53)^.

It is important to note that while Americans are among the highest global meat consumers, overall protein intake remains within the Acceptable Macronutrient Distribution Range^(54)^. Thus, substituting red and processed meats with other protein sources does not imply an increase in total protein intake but rather a realignment towards more healthful sources. These substitutions align with dietary recommendations aimed at reducing the intake of saturated fats and increasing the consumption of polyunsaturated fats and other beneficial nutrients^(26)^.

The findings of this study have important implications for public health. First, our results indicate that higher expenditures on red and processed meats are associated with increased overall food costs. Therefore, reducing the consumption of these meats could decrease food expenditures, which is particularly beneficial for low-income households that allocate a larger portion of their budget to food. Public health messaging should, therefore, emphasise the health and environmental benefits of reducing red and processed meat consumption and the potential economic benefits. Additionally, our study shows the possibility of substituting red and processed meats with alternatives like poultry and plant proteins without incurring additional costs. By promoting both reduction and substitution strategies, public health initiatives can help households improve their diets in ways that are both economically sustainable and aligned with nutritional guidelines.

However, it is important to note that not all protein substitutions are cost-neutral. Our results indicate that substituting meat with seafood high in *n*-3 fatty acids, which may have higher health benefits, was associated with increased food expenditures. This highlights the need for policies to enhance the affordability and accessibility of seafood high in *n*-3 fatty acids. Measures such as subsidies for *n*-3 rich seafood production and distribution, reductions in prices and taxes and expanded availability in low-income neighbourhoods could facilitate more equitable access to these healthy foods.

While our analysis primarily focused on protein substitutions, broader dietary modifications—such as reducing red and processed meat consumption while increasing the intake of under-consumed, nutrient-rich foods such as fruits, vegetables and whole grains—remain critical for improving public health outcomes^(25)^. Although these broader substitutions were beyond the scope of our current study, such dietary shifts align with current nutritional guidelines and hold substantial potential for improving overall public health. Future studies should assess these broader dietary modifications’ economic feasibility and implications.

This study’s strengths include using a nationally representative sample, the comprehensive measurement of food acquisitions and purchases made by all household members, adjusting energy intake and other confounders and estimating substitution effects of other protein sources. Food acquisitions were recorded over a 7-day period, which is more likely to reflect usual dietary intake, and most acquisition reports were done using scanners, reducing recall bias. Compared to other studies that estimate costs by imputing the retail value of foods and assuming that most foods are purchased at a retail store and consumed at home, this study uses households’ actual food expenditures at home and away from home. Additionally, the effects of purchasing red and processed meat were analysed separately, allowing for differentiation between two meat categories with distinct nutritional and cost profiles.

The main limitations are the cross-sectional design and the lack of information on food waste or individual-level food consumption patterns, which may affect the actual intake of foods. Because data were collected between April 2012 and January 2013, some seasonal variations in meat consumption may have been captured or missed. It is also possible that data collected in 2012–13 do not reflect the most recent purchasing trends. However, evidence suggests that increases in food prices over time have a more pronounced and negative effect on the consumption of other goods and services rather than an effect on the food budget, so the main insights from the results may still be relevant^(55)^.

Additionally, our study did not differentiate between the quality of purchased red or processed meats. Households of different income levels may purchase different quality meats, which could influence the overall cost implications. Future research should address this by examining the quality and types of meat purchased across different income groups.

Finally, this study did not consider other non-monetary costs that may be involved in making protein substitutions, such as time, skills and preferences. Cooking methods can also significantly affect both the nutritional quality and the cost of the food^(56)^. Future research should explore other barriers and facilitators influencing protein choices among different income groups and design more effective strategies to promote healthy eating habits.

In conclusion, this study showed that high red meat and processed meat consumption were associated with higher food expenditures in US households, especially for those with low income. This suggests that reducing these meats could result in cost savings. Substituting red or processed meat with poultry and plant proteins appeared cost-neutral, aligning with dietary guidelines recommending such substitutions. However, substituting meat with seafood, particularly varieties high in *n*-3 fatty acids, can increase food expenditures. Future public health interventions and policies should promote the substitution of red and processed meat with other protein sources that are comparable in cost while promoting the affordability and accessibility of seafood, particularly seafood high in *n*-3 fatty acids.

## Supporting information

Orta-Aleman et al. supplementary material Orta-Aleman et al. supplementary material

## Supplementary material

For supplementary material accompanying this paper visit https://doi.org/10.1017/S1368980024001897
