# Supplementary material for: Reduced red and processed meat consumption is associated with lower diet costs in US households: a national analysis of protein substitutions
Source: Public Health Nutr. 2024 Oct 10;27(1):e205. doi: 10.1017/S1368980024001897 (PMC11604326; doi:10.1017/S1368980024001897)
Supplement: Orta-Aleman et al. supplementary material [file S1368980024001897sup001.docx]

Appendix

Table S1. Red meat, processed meat, plant-protein, poultry and fish and seafood variables description.

| Variable | Components | % | Examples |
| --- | --- | --- | --- |
| *Total Processed Meat*  Cured/luncheon meat that has been preserved by smoking, curing, salting, and/or the addition of chemical preservatives | *Processed Red meat.*  Cured/luncheon meat made from beef or, pork preserved by smoking, curing, salting, and/or the addition of chemical preservatives | 83.8% | Pork bacon, beef frankfurters, beef sausages, etc. |
|  | *Processed White meat.*  Cured/luncheon meat poultry preserved by smoking, curing, salting, and/or the addition of chemical preservatives | 16.2% | Cold cuts and cured meats from turkey or chicken; chicken sausages, chicken bacon, etc. |
| *Total Red Meat*  Combination of ‘unprocessed red meat’ + ‘organ red meat’ + 'processed red meat'. Includes single ingredient items and mixed dishes | *Unprocessed red meat*  Beef, veal, pork, lamb, and game meat; excludes organ meat and cured meats. | 53.6% | Beef steak, ground beef, hamburger meat, pork chops, tacos with beef, etc. |
|  | *Organ red meat*  Organ meat from beef, veal, pork, lamb, game. | 0.4% | Beef liver, beef tongue, tripe. |
|  | *Processed Red meat.*  Cured/luncheon meat made from beef or pork. Preserved by smoking, curing, salting, and/or the addition of chemical preservatives | 46% | Pork bacon, beef frankfurters, beef sausages, etc. |
| *Total Plant proteins*  Combination of soy products, nuts, peanuts, seeds and legumes. | *Nuts, peanuts, and seeds.* | 32% | Peanut butter, almonds, peanuts, pistachio, cashew nuts, salted pecans, sesame seeds, etc. |
|  | *Legumes* | 61% | Baked beans, refried beans, hummus, etc. |
|  | *Soy products.* | 8% | Vegetarian burger, soy chips, tofu, soy yogurt, meatless bacon bits, etc. |
| Poultry (oz. eq.)  single ingredient items and mixed dishes from chicken, turkey, duck and bird game. | Unprocessed poultry | 98.5% | Chicken breast, chicken nuggets, turkey with gravy, chicken sandwich |
|  | Organ poultry | 0.1% | Pate, giblets |
|  | Processed poultry | 1.4% | Turkey deli meat, turkey bacon |
| Fish and seafood  Fresh, frozen or canned | High in omega-3 | 23.8% | Salmon, sardine, mackerel, cod, tuna, anchovies, etc |
|  | Low in omega-3 | 76.2% | Shrimp, catfish, tilapia, cod |

^a^ Each meat category included both single ingredient items (e.g., raw beef steak) and mixed dishes (e.g., pepperoni pizza). Some mixed dishes combine different kinds of meat. For mixed dishes, only the ounce equivalent proportion corresponding to each meat component was counted for the creation of the variables.

Table S2. Exponentiated coefficients of weekly household food expenditures by weighted quintile of weekly red and processed meat purchases

|  | Fully adjusted model^a^ | | | | | |
| --- | --- | --- | --- | --- | --- | --- |
|  | Weighted quintile of weekly **red meat purchases** per AME | | | Weighted quintile of weekly **processed meat** purchases per AME | | |
|  | $e^{\beta}$ | (95% CI) | $e^{\beta}$ | | (95% CI) |  |
| Q1 | 1 | [Ref] | 1 | | [Ref] |  |
| Q2 | 1.17 | (1.04, 1.3) | 1.06 | | (0.95, 1.19) |  |
| Q3 | 1.29 | (1.15, 1.46) | 1.24 | | (1.09, 1.39) |  |
| Q4 | 1.48 | (1.29, 1.7) | 1.25 | | (1.10, 1.42) |  |
| Q5 | 1.51 | (1.27, 1.78) | 1.33 | | (1.16, 1.53) |  |

^a.^ Fully adjusted regression estimates from generalized linear model with gamma distribution and log link, incorporating FoodAPS strata and sampling weights, and adjusted for household size, rurality, household income to poverty ratio, geographical area, number of children in the household, number of adult members over 60 in the household, SNAP participation, proportion of food-away-from-home vs food-at-home, weekly energy per AME; and primary respondent demographic characteristics including sex, age, race/ethnicity, education, and being married.
AME: Adult Male Equivalent. CI: Confidence Interval. HH: Household. SNAP: Supplemental Nutrition Assistance Program.. Q: quintile. Ref: Reference.
